# Are estimands being correctly used? A review of UK research protocols

**DOI:** 10.1186/s13063-025-08991-8

**Published:** 2025-08-26

**Authors:** Timothy P. Clark, Richard H. Wicentowski, Suzie Cro, Matthew R. Sydes, Brennan C. Kahan

**Affiliations:** 1 MRC Clinical Trials Unit at UCL, Institute of Clinical Trials and Methodology, UCL, 90 High Holborn, 2 Floor, London, WC1V 6LJ UK; 2 Computer Science Department, Swarthmore College, Swarthmore, PA USA; 3 Imperial Clinical Trials Unit, Imperial College London, London, UK; 4 Data for R&D, Transformation Directorate, NHS England, London, UK

**Keywords:** Estimands, ICH E9(R1) Addendum, Transparency, Clarity, Research question, Treatment effect

## Abstract

**Background:**

The use of estimands in clinical trials was formalised with the adoption of the final International Conference on Harmonisation E9 Addendum on Estimands and Sensitivity Analysis in Clinical Trials (ICH E9(R1) Addendum) in November 2019. The declared objective of the ICH E9(R1) Addendum is to bring clarity and transparency to the research question of interest. For this to be achieved, the estimand must be described in accordance with the requirements of the ICH E9(R1) Addendum so that the target treatment effect is clear to all stakeholders. Previous reviews of publications and published protocols have found that few trials explicitly defined the primary estimand. To obtain a more complete picture of how the use of estimands has changed over time, whether trials are using estimands correctly (i.e. correctly defining the five attributes of an estimand), and which strategies are being used to handle intercurrent events, we obtained access to an extensive database of original research protocols (*n* = 29,212) submitted to the United Kingdom’s Health Research Authority, which oversees ethical review of clinical trials.

**Methods:**

Protocols were eligible for review if they included the term ‘estimand’ and attempted to define at least one attribute of the primary estimand. For eligible protocols, we extracted information on trial characteristics such as whether the trial was randomized and the therapeutic area, as well as whether the estimand attributes used for the primary outcome were correctly defined, and which strategies were used to handle intercurrent events.

**Results:**

We found that the number of protocols defining a primary estimand increased starkly with publication of the ICH E9(R1) Addendum (approximately 3 protocols/year pre-ICH E9(R1) Addendum vs. 18 protocols / year during the consultation period vs. 23 protocols in the year following the adoption of the ICH E9(R1) Addendum). However, the description of the primary estimand was suboptimal; many protocols failed to mention specific attributes (such as population or treatment conditions) in the estimand description, and many protocols incorrectly defined estimand attributes (e.g. by describing the estimand population based on their analysis population).

**Conclusions:**

Although release of the ICH E9(R1) Addendum has dramatically increased the use of estimands in clinical trials, their reporting is suboptimal. There is still work to be done to ensure estimands reach their full potential in bringing clarity and focus to research questions.

**Supplementary Information:**

The online version contains supplementary material available at 10.1186/s13063-025-08991-8.

## Background

Estimands have been discussed in the statistical literature for several decades, but their use in clinical trials was only formalised with the adoption of the final International Conference on Harmonisation E9 Addendum on Estimands and Sensitivity Analysis in Clinical Trials (ICH E9(R1) Addendum) in November 2019 [1–5]. An estimand is a precise definition of the treatment effect the investigator intends to estimate in the trial. As per the ICH E9(R1) Addendum, estimands encompass five attributes that must be defined: treatment condition(s), population, endpoint (outcome measure), population-level summary measure, and specification of how intercurrent events (ICEs) such as treatment discontinuation or mortality are to be handled. Table 1 provides an overview of estimands. The ICH E9(R1) Addendum as well as the role of estimands have been discussed extensively elsewhere [6–17].

**Table 1:** Overview of estimands

• Defining an estimand entails defining five attributes: treatment condition(s), population, endpoint (outcome measure), population-level summary measure, and strategy for handling ICEs • ICH E9 (R1) addendum suggests five potential strategies to address ICEs: 1. Treatment policy strategy: occurrence of the ICE is considered irrelevant, and interest lies in the treatment effect regardless of whether the ICE occurred. Note that this approach cannot be used for truncating events, e.g. where a variable cannot be measured due to death 2. Hypothetical strategy: treatment effect under a scenario where the intercurrent event(s) would not occur 3. Composite strategy: ICE is incorporated into the outcome definition, for instance, patients who experience the ICE are classed as a non-responders 4. While-on-treatment strategy: response to treatment before occurrence of intercurrent event(s) is of interest 5. Principal stratum: treatment effect in the “principal stratum” of interest (e.g. the population of patients who would not experience the ICE under either treatment assignment)	

*ICH* International Conference on Harmonisation, *ICE* intercurrent event

The aim of the estimand framework is to improve the clarity and transparency of clinical trials [9]. Hence a complete description of the estimand is required so that the target treatment effect being estimated is clear to all stakeholders.

Previous reviews of publications of randomised clinical trials and published protocols have found that few trials explicitly define the estimand [18, 19]. A systematic review of 255 randomized trials published in six leading general medical journals found that no trials clearly stated all the attributes of the estimand [18]. A review of 50 research protocols published in *Trials* and *BMJ Open*, found that none explicitly stated the estimand for the primary outcome [19].

The limitation of these reviews is that they depend on the information that is released in the publication domain, so may be affected by publication bias [20]. In any event, little is known about the use of estimands and how well they are described in research protocols. We aimed to fill this gap by obtaining permission to review of the use of estimands in original research protocols submitted to the United Kingdom’s Health Research Authority (HRA), which oversees national ethical review of health research conducted within the National Health Service (NHS), during the period directly before and after the adoption of the ICH E9(R1) Addendum. The HRA database aims to include all clinical trials conducted in the United Kingdom, e.g. trials of investigational medicinal products, medical devices and surgical interventions [21]. It is extensive as the UK is highly research active with around 1,000 randomised clinical trials submitted per year for approval [22].

### Methods

We used the methods previously developed by the authors (TC, RW, MS), to interrogate the HRA database of application forms, which contain very detailed information taken directly from the original research protocol [22]. The HRA database contained applications for clinical trials (except Phase I healthy volunteer trials) submitted during the period January 2011 to December 2020. We identified all trials where the term ‘estimand’ (singular or plural) was used in the application form.

Protocols were eligible for review if they included the term ‘estimand’ and attempted to define at least one attribute of the primary estimand. For eligible protocols, we extracted baseline characteristics to help characterise the types of trials included in this review, such as protocol date, whether the trial was randomized, number of treatment arms, therapeutic area, etc. To address our primary objectives, we also extracted information on how well estimand attributes for the primary outcome were described, which ICEs were identified, and which strategies were used to handle ICEs. We designed a data extraction form to capture relevant information for each eligible protocol (Supplemental File 1).

We assessed if the estimand attributes were correctly defined (see references 6–8 for articles which contain examples of correctly defined estimands). For the attributes, treatment condition(s), population, endpoint (outcome measure) and population-level summary measure, this was summarised as:

- ‘Yes’ (correctly defined)
- ‘No (did not attempt to define)’
- ‘No (not correctly defined)’ or
- ‘Unclear’.

If the response was ‘No (not correctly defined)’ or ‘Unclear’ then the reason was documented. These attributes could be incorrectly defined if, for example, some essential information was missing (e.g. medication dose or frequency not described, or the time-frame of the endpoint not listed), or if they were defined as, or based on, the trial’s analysis methods (e.g. the population was defined in terms of the analysis population).

For ICEs the same approach was used. The handling of an ICE was correctly defined if one of the five strategies from ICH-E9(R1) were listed. If one of the five strategies was not explicitly listed, but the method of handling the ICE was described in a way that clearly aligned with one of the strategies (e.g. “The estimand addressed the effect of the intervention regardless of treatment discontinuation” or “The estimand addressed the effect of the intervention if no patients discontinued their assigned treatment”), then this was classified as being correctly defined. The handling of an ICE was typically incorrectly defined if its handling was defined based on the trial’s methods, for instance if the principal stratum population was defined based on the analysis population (e.g. “the principal stratum population was comprised of patients who received at least 50% of their assigned treatment”).

If the hypothetical strategy was used then we checked to see how the envisaged hypothetical scenario would occur, e.g., for treatment discontinuation due to adverse events did the authors envisage participants continuing treatment despite the adverse events? For instance, by providing additional treatments to manage symptoms or side effects, such as anti-nausea medication for chemotherapy-induced nausea.

Initially, two reviewers (TC and BK) evaluated each protocol independently, and resolved disagreements through discussion. After approximately 60% of the protocols had been evaluated, the concordance between the reviewers was over 90% so it was decided to end the independent review and have one reviewer (TC) complete the remaining extractions.

## Results

The full results of the review appear in Supplementary File 2. We identified 122 protocols (< 1% of the trials in the HRA database [*n* = 29,212]) with the term “estimand” or “estimands”, of which 81 (66%) were eligible for review, i.e., attempted to define at least one attribute of the estimand (Supplementary File 2, Table 1).

### Characteristics of eligible protocols

Most trials defining estimands were randomised (80/81 [99%]), had 2 treatment arms (52/81 [64%]), were phase 3 (61/81 [75%]), assessed pharmacological interventions (81/81 [100%]), aimed to demonstrate superiority of the test over the control invention (67/81 [83%]) and were industry sponsored (76/81 [94%]; Table 2 and Supplement File 2, Table 2). Notably, no non-industry sponsored trials were identified – the sponsorship of the 5 (6%) other studies was not given.

**Table 2:** Most commonly reported characteristics of eligible studies (*n* = 81)*

**Study Characteristic**	n (%)
Randomized Trial n (%)	80 (98.8)
Number Treatment Arms	
2	52 (64.2)
3	20 (24.7)
Clinical Phase	
2	17 (21.0)
3	61 (75.3)
Pharmacological intervention	81 (100.00)
Trial Type	
Non-inferiority	8 (9.9)
Superiority	67 (82.7)
Commercial Status	
Industry sponsored	76 (93.8)

^*^81 protocols were eligible for review. Only selected results are shown in some categories, so sub-categories may not sum to 81. Full results are given in Supplement File 2, Table 2

The median sample size was 663 (interquartile range: 159—800; Supplement File 2, Table 2).

The most common therapeutic areas were neurology (14/81 [17%]), endocrinology (13/81 [16%]) and musculo-skeletal (11/81 [14%]) (Supplement File 2, Table 3).

**Table 3:** Primary estimand attributes (*n* = 81)

Population	n (%)
Defined correctly	29 (35.8)
No (did not attempt to define)	17 (21.0)
No (not correctly defined)^**a**^	35 (43.2)
Treatment	
Defined correctly	36 (44.4)
No (did not attempt to define)	45 (55.6)
Endpoint	
Defined correctly	59 (72.8)
No (did not attempt to define)	21 (25.9)
No (not correctly defined)^**b**^	1 (1.2)
Summary measure	
Defined correctly	44 (54.3)
No (did not attempt to define)	37 (45.7)

^a^Estimand population defined as analysis population

^b^Time point for assessment not given

### Impact of ICH E9(R1) Addendum on use of estimands in eligible protocols

We found that use of estimands increased starkly with the publication of the Addendum: approximately 3 protocols/year (21/22,396 [0.1%] applications submitted during the 7-year period before the publication of the ICH E9(R1) Addendum [January 2011 to August 2017]); 18 protocols/year (37/5,733 [0.6%] applications submitted during the 2-year consultation period [September 2017 to November 2019]); and 23/1,083 (2.1%) protocols submitted in the 1-year following the adoption of the final ICH E9(R1) Addendum (Supplement File 2, Table 5).

### Reporting of estimand attributes in eligible protocols

Although all eligible protocols tried to describe their primary estimand, many protocols omitted information on one or more estimand attributes in their description. For example, 21% (17/81) did not attempt to describe the population attribute, 56% (45/81) the treatment condition(s) attribute, 26% (21/81) the endpoint attribute and 46% (37/81) the summary measure attribute (Table 3 and Supplementary File 2, Table 4).

**Table 4:** ICEs defined in study protocols (*n* = 81)

ICE	n (%)
Treatment non-adherence / no reason specified	68 (84.0)
Treatment non-adherence / due to adverse event	7 (8.6)
Treatment non-adherence / not due to an adverse event	5 (6.2)
Use of rescue therapy	29 (35.8)
Treatment switching	3 (3.7)
Death	2 (2.5)
Other terminal event	1 (1.2)
Other ICE	18 (22.2)

ICE intercurrent event

**Table 5:** Other ICEs

ICE	n (%)
Did not receive study drug/study medication	14/20 (70.0)
Dose reduction or suspension of treatment	1/20 (5.0)
Ineligibility for follow-on study treatment	1/20 (5.0)
Participants who start a protocol prohibited medication/therapy	2/20 (10.0)
Change in background medication	1/20 (5.0)
Need for surgery	1/20 (5.0)

*ICE* intercurrent event

^*^2 studies had 2 Other ICEs (*n* = 20)

Many protocols also described the estimand attributes incorrectly. For example, 43% (35/81) of protocols incorrectly described the estimand population based on which patients would be included in the statistical analysis, rather than which patients were targeted by their clinical question (Table 3 and Supplementary File 2, Table 4). Also, rather surprisingly, 25.9% (21/81) of protocols did not attempt to define the endpoint within the estimand and 45.7% (37/81) did not attempt to define the summary measure.

As discussed above, we also analysed the primary estimand attributes by time period: prior to release of the draft ICH E9(R1) Addendum (before September 2017); during the consultation period (September 2017 to November 2019); and after adoption (December 2019 to December 2020)—Supplementary File 2, Table 5. Although, the number of protocols reporting the use of estimands increased after the publication of the guidance, the proportion of protocols that correctly defined their estimand did not change appreciably.

### Intercurrent events in eligible protocols

Almost 1 in 10 protocols (8/81 [10%]) incorrectly listed at least one ICE in their estimand which did not meet the standard definition of an intercurrent event (Supplementary File 2, Table 6). For 6 out of the 8 [75%] protocols, this involved incorrectly listing missing outcome data as an ICE.

**Table 6:** Strategy for handling of treatment non-adherence with no reason specified

ICE strategy	n (%)
Treatment policy	35/62 (56.5)
Hypothetical	15/62 (24.2)
Composite	10/62 (16.1)
Principal stratum	0/62 (0.0)
While-on-treatment	2/62 (3.2)
Hypothetical scenario^a^	
Given	4/15 (26.7)
Not given	11/15 (73.3)

*ICE* intercurrent event

^a^Did the authors state *how* the envisaged hypothetical scenario would occur, e.g., for treatment discontinuation due to adverse events did the authors envisage participants continuing treatment *despite* the adverse events, or something else?

The most defined ICE was treatment non-adherence / discontinuation where no reason was specified (68/81 [84%]), with use of rescue therapy the next most common (29/81 [36%]; Table 4 and Supplementary File 2 Tables 7–13). The other 5 pre-specified ICEs were less frequently defined (< 10% of protocols). Perhaps most surprisingly, discontinuation due to an adverse event, which is a frequent occurrence in most clinical trials, was only defined in 7 (9%) of protocols.

**Table 7:** Strategy for handling use of rescue medication (*n* = 81)

ICE strategy	n (%)
Treatment policy	8/24 (33.3)
Hypothetical	5/24 (20.8)
Composite	11/24 (45.8)
Principal stratum	0/24 (0.0)
While-on-treatment	0/24 (0.0)
Hypothetical scenario^a^	
Given	0/5 (0.0)
Not given	5/5 (100.0)

*ICE* intercurrent event

^a^Did the authors state *how* the envisaged hypothetical scenario would occur, e.g., for treatment discontinuation due to adverse events did the authors envisage participants continuing treatment *despite* the adverse events, or something else?

There were 20 “Other ICE” defined in 18 protocols (Table 5 and Supplementary File 2 Table 14). Non-receipt of study drug/study medication (14/20 [70%]) was the most defined “Other ICE”.

Treatment policy (35/62 [57%]) and hypothetical (15/62 [24%]) were the most common strategies to handle treatment non-adherence with no reason specified (Table 6 and Supplementary File 2 Table 7).

However, most trials using a hypothetical strategy (11/15 [73%]) did not specify the mechanisms behind the hypothetical setting of interest.

Composite (11/24 [46%]), treatment policy (8/24 [33%]) and hypothetical (5/24 [21%]) were the strategies used to handle use of rescue therapy (Table 7 and Supplementary File 2 Table 10). None of the trials using a hypothetical strategy specified the mechanism.

## Discussion

To our knowledge this is the most extensive review of the use of estimands in clinical trials that has been undertaken. We focussed on the period directly before and after the adoption of the ICH E9(R1) Addendum on Estimands and Sensitivity Analysis in Clinical Trials. While we found that the number of protocols defining estimands increased markedly following the release of the ICH E9 Addendum (approximately 3 protocols/year pre-ICH E9 Addendum. to 23 protocols in the year following the adoption of the ICH E9 Addendum) the overall number was still relatively low representing < 1% of the trials per year in the HRA database. The vast majority of eligible trials were industry sponsored and evaluated pharmacological interventions, which is not surprising as the ICH E9(R1) Addendum is an ICH guideline, which are primarily aimed at industry.

Of note, even though the publication of the draft ICH E9 Addendum resulted in an increased use of estimands, their reporting was suboptimal. For instance, authors frequently failed to define all attributes correctly. In particular, the estimand population was often defined in terms of the analysis population, i.e. which patients would be included in the statistical analysis. e.g. the modified intention-to-treat (mITT) set, which excludes patients on the basis of non-receipt of study drug or some other event. However, this approach fails to recognise that the criteria used to exclude patients from the analysis set are ICEs, which should be defined in the estimand [23].

There was also a lack of differentiation in terms of ICEs with “Treatment discontinuation / non-adherence where no reason specified” the most commonly defined. Being clear about the reason for treatment discontinuation is important as it allows those evaluating the trial to understand whether an appropriate strategy was used for the ICEs. For instance, a hypothetical strategy may pose challenges when applied to treatment discontinuation for toxicity or adverse events, so giving the exact reason for treatment discontinuation helps understand the clinical relevance of the estimand [5]. This is because the hypothetical strategy assumes that patients would have continued the treatment despite experiencing an adverse event, which may not be clinically meaningful, as adverse events are often a direct consequence of the treatment [24].

It is therefore crucial for the investigator to explain how the hypothetical scenario would occur [8]. For example, is it envisaged that subjects would be given additional medication to manage symptoms so that they can continue treatment, or would the dose of the investigational drug be reduced to help mitigate adverse events? It is clear to see that these two scenarios are quite different and could lead to different outcomes.

Unfortunately, most trials using a hypothetical strategy did not specify how the hypothetical scenario would occur, which hinders the proper interpretation of the clinical relevance and validity of the hypothetical strategy [8].

A strength of our review is that we had access to a very extensive database of research protocols unaffected by publication bias. A limitation of our review is that we had access to research protocols submitted only until the year following the adoption of the final ICH E9 Addendum, i.e. 2020. Our definition of correctly using estimands considered whether all 5 estimand attributes were fully stated, with no essential information missing and not incorrectly based on methods for estimation rather than targeted. We were unable to assess whether the presented estimands aligned with the precise research question the investigators intended to be addressed.

Our review of protocols submitted to HRA for ethical review suggests that although estimands are being used more widely since the publication of the ICH E9 Addendum, the proportion of protocols that correctly defined the primary estimand did not change appreciably. Hence, the clarity that estimands provide in terms of the treatment effect being estimated was often lost.

If the estimand framework is going to deliver greater transparency and clarity to the research question then estimands must not only be used when planning the clinical trial, but also correctly defined. To achieve this, investigators need to be trained either through in-person workshops, webinars or the many tutorial articles that have been published on this topic [8, 25–27]. Furthermore, both ICH M11 and TransCelerate protocol templates provide guidance on the use of estimands in clinical trials by incorporating structured sections for their definition and integration with study objectives and endpoints [28, 29]. Finally, regulatory authorities must also play a leading role in ensuring that estimands are correctly defined and consistently applied in clinical trials.

## Conclusion

We found an increase in the use of estimands in clinical research in the UK following the publication of the ICH E9 Addendum. The reporting of the estimand was often suboptimal suggesting that more work to promote good practice is required.

## Authors’ contributors

TC had the original idea, designed the extraction form, reviewed the protocols, analysed the data and wrote the first draft. BK designed the extraction form, reviewed the protocols, revised the paper and approved the final version. RHW: collected the data and reviewed and approved final manuscript. SC designed the extraction form and reviewed and approved final manuscript. MRS: collected data, reviewed and approved final manuscript. TC is guarantor.

## Supplementary Information

Supplementary Material 1.

Supplementary Material 2.

## Data Availability

The data used in this analysis were obtained from the Health Research Authority under a confidentiality agreement and is not under the researcher’s gift to share. Applications for these data or expanded data must involve the HRA.

## Abbreviations

HRA: Health Research Authority
ICE: Intercurrent events
ICH: International Conference on Harmonisation
mITT: Modified intention-to-treat
NHS: National Health Service
UK: United Kingdom
