# Supplementary material for: Are estimands being correctly used? A review of UK research protocols
Source: Trials. 2025 Aug 26;26:310. doi: 10.1186/s13063-025-08991-8 (PMC12379330; doi:10.1186/s13063-025-08991-8)
Supplement: Supplementary file 1 — Supplementary Material 1. [file 13063_2025_8991_MOESM1_ESM.docx]

**Data extract form v1.0**

**Section 1: Study identifiers**

| **Question** | **Answer** |
| --- | --- |
| IRAS project ID |  |
| Name of extractor |  |

**Section 2: Eligibility**

| **Question** | **Answer** |
| --- | --- |
| Does the protocol attempt to define the estimand for the study’s primary outcome? ^a^ | Yes ^b^  No (labelled/named the estimand, but did not define)^c^  No (no attempt to define)^d^  Unclear |

^a^If more than one outcome is designated as primary, use the first outcome listed. If more than one estimand is designated as primary, use the first one listed for the primary outcome.

^b^ Defined this way if investigators attempted to define at least one attribute of the estimand (e.g. if they stated that all intercurrent events [ICEs] were handled using a treatment policy strategy)

^c^ Defined this way if investigators labelled or named the estimand (e.g. “we used a trial product estimand”) without explicitly defining any attributes

^d^ Defined this way if investigators did not attempt to define any attributes or label the estimand

***For any answer but “yes”, the protocol is not eligible, and the rest of the questions do not need to be filled out***

**Section 3: Trial characteristics**

| **Characteristic** | **Answer** |
| --- | --- |
| Protocol date | mm/yyyy |
| Randomised trial? | Yes/no/unclear |
| Number of treatment arms |  |
| Clinical phase (select one) | - 1 - 1/2 - 2 - 2/3 - 3 - 4 - not stated |
| Therapeutic area | - Allergy/Immunology (e.g., allergic rhinitis). - Cardiovascular/Vascular disease (e.g., angina, hypertension) - Dental/Maxillofacial Surgery - Dermatology (e.g., acne, psoriasis) - Endocrinology (e.g., diabetes, obesity, weight loss) - Gastroenterology (e.g., ulcerative colitis, Crohn’s Disease) - Haematology (e.g., iron deficiency anaemia, haemophilia) - Hepatology (e.g., Non-alcoholic steatohepatitis [NASH]) - Infectious diseases (e.g., influenza, urinary tract infections) - Musculo-skeletal (e.g., osteoarthritis, rheumatoid arthritis) - Nephrology / Urology (e.g., chronic kidney disease) - Neurology (e.g., Parkinson’s, Alzheimer’s) - Oncology (e.g., breast cancer, leukaemia) - Ophthalmology (e.g., age-related macular degeneration) - Otolaryngology (e.g., otitis media) - Psychiatry / Psychology (e.g., depression, insomnia) - Respiratory (e.g., asthma, smoking cessation, COPD) - Surgery - Other - Unclear |
| Intervention type | - Pharmacologic - Surgical - Psychosocial/behavioural/educational - Multiple intervention types - Other - Unclear |
| Type of trial (select one) | - Superiority - Equivalence - Non-inferiority - Other - Unclear |
| If **equivalence**, is it a bioequivalence study? | - Yes - No - Unclear |
| Sample size |  |
| Commercial status (select one) | - Industry sponsored - Non-industry sponsored - Unclear |

**Section 4: Primary estimand for primary outcome* (except intercurrent events [ICEs] – see Section 6)**

| **Question** | **Answer** |
| --- | --- |
| Was the **Population** aspect correctly defined? | - Yes - No (did not attempt to define) - No (not correctly defined) - Unclear |
| If **unclear**, state why (*free text*) |  |
| If **no (not correctly defined)**, state why (*free text*) |  |
| Was the **Treatment condition(s)** aspect correctly defined | - Yes - No (did not attempt to define) - No (not correctly defined) - Unclear |
| If **unclear**, state why (*free text*) |  |
| If **no (not correctly defined)**, state why (*free text*) |  |
| Was the **Endpoint** aspect correctly defined | - Yes - No (did not attempt to define) - No (not correctly defined) - Unclear |
| If **unclear**, state why (*free text*) |  |
| If **no (not correctly defined)**, state why (*free text*) |  |
| Was the **Summary measure** aspect correctly defined | - Yes - No (did not attempt to define) - No (not correctly defined) - Unclear |
| If **unclear**, state why (*free text*) |  |
| If **no (not correctly defined)**, state why (*free text*) |  |

*If more than one outcome is designated as primary, use the first outcome listed. If more than one estimand is designated as primary, use the first one listed for the primary outcome.

**Section 5: Were any incorrect ICEs specified?**

| **Question** | **Answer** |
| --- | --- |
| Was anything specified as an ICE which did not meet the definition of an ICE?* (e.g. missing data, study withdrawal/discontinuation, etc) | - Yes - No - Unclear |
| If **yes**, what was it? (*select all that apply)* | - Missing data - Study withdrawal - Other |
| If **other**, describe (*free text)* |  |

*An ICE is a post-randomisation/post-baseline event which affects the interpretation or existence of outcome data. Common examples are things which affect receipt of treatment (treatment discontinuation, treatment switching, failure to start treatment, incorrect dose of treatment, etc), or which affect existence of outcome data (e.g. death if it’s not defined as part of the outcome). Things such as missing data, loss-to-follow-up, or study withdrawal are **not** ICEs

**Section 6: Handling of ICE’s for primary estimand**

| **Question** | **Answer** |
| --- | --- |
| **ICE1: Treatment non-adherence/ discontinuation where no reason specified** |  |
| Was this listed as one of the ICEs? | Yes/no/unclear |
| If **yes**, was its handling correctly defined?^a^ | - Yes - No (did not attempt to define) - No (not correctly defined) - Unclear |
| If **unclear**, state why (*free text*) |  |
| If **no (not correctly defined)**, state why (*free text*) |  |
| If **yes**, what strategy was used? | - Treatment policy - Hypothetical - Composite - Principal stratum - While-on-treatment |
| If **hypothetical**, was the mechanism leading to the envisaged hypothetical scenario given^b^? | Yes/no/unclear |
| **ICE2: Treatment non-adherence/ discontinuation due to adverse event** |  |
| Was this listed as one of the ICEs? | Yes/no/unclear |
| If **yes**, was its handling correctly defined?^a^ | - Yes - No (did not attempt to define) - No (not correctly defined) - Unclear |
| If **unclear**, state why (*free text*) |  |
| If **no (not correctly defined)**, state why (*free text*) |  |
| If **yes**, what strategy was used? | - Treatment policy - Hypothetical - Composite - Principal stratum - While-on-treatment |
| If **hypothetical**, was the mechanism leading to the envisaged hypothetical scenario given^b^? | Yes/no/unclear |
| **ICE3: Treatment non-adherence/ discontinuation due to a specified reason other than adverse event** (**If multiple ICEs given based on different reasons, include one in this section, and the rest under the “Other ICE” section below) |  |
| Was this listed as one of the ICEs? | Yes/no/unclear |
| If **yes**, was its handling correctly defined?^a^ | - Yes - No (did not attempt to define) - No (not correctly defined) - Unclear |
| If **unclear**, state why (*free text*) |  |
| If **no (not correctly defined)**, state why (*free text*) |  |
| If **yes**, what strategy was used? | - Treatment policy - Hypothetical - Composite - Principal stratum - While-on-treatment |
| If **hypothetical**, was the mechanism leading to the envisaged hypothetical scenario given^b^? | Yes/no/unclear |
| **ICE4: Use of rescue therapy** |  |
| Was this listed as one of the ICEs? | Yes/no/unclear |
| If **yes**, was its handling correctly defined? | - Yes - No (did not attempt to define) - No (not correctly defined) - Unclear |
| If **unclear**, state why (*free text*) |  |
| If **no (not correctly defined)**, state why (*free text*) |  |
| If **yes**, what strategy was used? | - Treatment policy - Hypothetical - Composite - Principal stratum - While-on-treatment |
| If **hypothetical**, was the mechanism leading to the envisaged hypothetical scenario given^b^? | Yes/no/unclear |
| **ICE5: Treatment switching** |  |
| Was this listed as one of the ICEs? | Yes/no/unclear |
| If **yes**, was its handling correctly defined?^a^ | - Yes - No (did not attempt to define) - No (not correctly defined) - Unclear |
| If **unclear**, state why (*free text*) |  |
| If **no (not correctly defined)**, state why (*free text*) |  |
| If **yes**, what strategy was used? | - Treatment policy - Hypothetical - Composite - Principal stratum - While-on-treatment |
| If **hypothetical**, was the mechanism leading to the envisaged hypothetical scenario given^b^? | Yes/no/unclear |
| **ICE6: Death** |  |
| Was this listed as one of the ICEs? | Yes/no/unclear |
| If **yes**, was its handling correctly defined?^a^ | - Yes - No (did not attempt to define) - No (not correctly defined) - Unclear |
| If **unclear**, state why (*free text*) |  |
| If **no (not correctly defined)**, state why (*free text*) |  |
| If **yes**, what strategy was used? | - Hypothetical - Composite - Principal stratum - While-alive |
| If **hypothetical**, was the mechanism leading to the envisaged hypothetical scenario given^b^? | Yes/no/unclear |
| **ICE7: Other terminal event^c^ (events which affect existence of outcomes)** |  |
| Was this listed as one of the ICEs? | Yes/no/unclear |
| If **yes**, was its handling correctly defined?^a^ | - Yes - No (did not attempt to define) - No (not correctly defined) - Unclear |
| If **unclear**, state why (*free text*) |  |
| If **no (not correctly defined)**, state why (*free text*) |  |
| If **yes**, what strategy was used? | - Hypothetical - Composite - Principal stratum - While-on-treatment |
| If **hypothetical**, was the mechanism leading to the envisaged hypothetical scenario given^b^? | Yes/no/unclear |
| **ICE8: Other ICE 1** |  |
| Description of ICE (*free text*) |  |
| Was this listed as one of the ICEs? | Yes/no/unclear |
| If **yes**, was its handling correctly defined?^a^ | - Yes - No (did not attempt to define) - No (not correctly defined) - Unclear |
| If **unclear**, state why (*free text*) |  |
| If **no (not correctly defined)**, state why (*free text*) |  |
| If **yes**, what strategy was used? | - Treatment policy - Hypothetical - Composite - Principal stratum - While-on-treatment |
| If **hypothetical**, was the mechanism leading to the envisaged hypothetical scenario given^b^? | Yes/no/unclear |
| **Copy and paste rows for “Other ICE” as many times as needed in order to capture all other ICEs** |  |

^a^ The handling of an ICE will generally be correctly defined if one of the five strategies from ICH-E9(R1) are listed to handle it. It will typically be incorrectly defined if its handling is defined based on the trial’s methods, for instance if the principal stratum population is defined based on the analysis population (e.g. “the principal stratum population was comprised of patients who received at least 50% of their assigned treatment”).

^b^Did the authors state *how* the envisaged hypothetical scenario would occur, e.g., for treatment discontinuation due to adverse events did the authors envisage participants continuing treatment *despite* the adverse events, or something else?

^c^ Terminal events are those which prevent the existence of the outcome. Examples include limb amputation when the outcome is a limb pain score at 1 year, or miscarriage when the outcome is neonatal birthweight. See ICH-E9(R1) for further explanation of terminal events.
