# Supplementary material for: Are estimands being correctly used? A review of UK research protocols
Source: Trials. 2025 Aug 26;26:310. doi: 10.1186/s13063-025-08991-8 (PMC12379330; doi:10.1186/s13063-025-08991-8)
Supplement: Supplementary file 2 — Supplementary Material 2. [file 13063_2025_8991_MOESM2_ESM.docx]

Table 1: Eligibility (n=122)

| Is the primary estimand defined? n (%) |
| --- |
| No (labelled/named the estimand, but did not define) ^a^ 7 (5.7) |
| No (no attempt to define) ^b^ 34 (27.9) |
| Yes ^c^ 81 (66.4) |

^a^ Defined this way if investigators labelled or named the estimand (e.g. “we used a trial product estimand”) without explicitly defining any attributes

^b^ Defined this way if investigators did not attempt to define any attributes or label the estimand

^c^ Defined this way if investigators attempted to define at least one attribute of the estimand (e.g. if they stated that all intercurrent events were handled using a treatment policy strategy)

Table 2: Characteristics of eligible studies (n=81)

| Randomized Trial? n (%)* |
| --- |
| No 1 (1.2) |
| Yes 80 (98.8) |
| Number Treatment Arms n (%) |
| 1 1 (1.2) |
| 2 52 (64.2) |
| 3 20 (24.7) |
| 4 8 (9.9) |
| Clinical Phase |
| 1 1 (1.2) |
| 1/2 1 (1.2) |
| 2 17 (21.0) |
| 3 61 (75.3) |
| 4 1 (1.2) |
| Intervention Type |
| Pharmacologic 81 (100.00) |
| Type of Trial |
| Non-inferiority 8 (9.9) |
| Superiority 67 (82.7) |
| Other 6 (7.4)   - Evaluate safety 3 (50.0) - Dose-response relationship 2 (33.33) - Single-arm Phase IIa 1 (16.67) |

Table 2 (cont.): Trial Characteristics of eligible studies (n=81)

| Sample Size |
| --- |
| Median (IQR) 662.8 (159.0-800.0) |
| Commercial Status |
| Industry sponsored 76 (93.8) |
| Unclear 5 (6.2) |

*except for sample size where the median and interquartile range (IQR) are listed

Table 3: Therapeutic Area (≥3 occurrences) of eligible studies (n=81)

| Therapeutic Area n (%) |
| --- |
| Allergy/Immunology (e.g., allergic rhinitis) 4 (4.9) |
| Cardiovascular/Vascular disease (e.g., angina, hypertension) 4 (4.9) |
| Dermatology (e.g., acne, psoriasis) 7 (8.6) |
| Endocrinology (e.g., diabetes, obesity, weight loss) 13 (16.1) |
| Hepatology (e.g., Non-alcoholic steatohepatitis [NASH]) 6 (7.4) |
| Infectious diseases (e.g., influenza, urinary tract infections) 4 (4.9) |
| Musculo-skeletal (e.g., osteoarthritis, rheumatoid arthritis) 11 (13.6) |
| Nephrology / Urology (e.g., chronic kidney disease) 3 (3.7) |
| Neurology (e.g., Parkinson’s, Alzheimer’s) 14 (17.3) |
| Psychiatry / Psychology (e.g., depression, insomnia) 3 (3.7) |
| Respiratory (e.g., asthma, smoking cessation, COPD) 5 (6.2) |
| Other 7 (8.6) |

Table 4: Primary Estimand Attributes (n=81)

| Population n (%) |
| --- |
| Was the Population correctly defined? |
| Yes 29 (35.8) |
| No (did not attempt to define) 17 (21.0) |
| No (not correctly defined) 35 (43.2) |
| Unclear 0 (0.0) |
| If no (not correctly defined), why? |
| Estimand population defined as analysis population 35/35 (100.0) |
| Treatment |
| Was the Treatment Condition(s) correctly defined? |
| Yes 36 (44.4) |
| No (did not attempt to define) 45 (55.6) |
| No (not correctly defined) 0 (0.0) |
| Unclear 0 (0.0) |
| Endpoint |
| Was the Endpoint correctly defined? |
| Yes 59 (72.8) |
| No (did not attempt to define) 21 (25.9) |
| No (not correctly defined) 1 (1.2) |
| Unclear 0 (0.00) |
| If no (not correctly defined), why? |
| Time point for assessment not given 1/1 (100.0) |
| Summary measure |
| Was the Summary Measure correctly defined? |
| Yes 44 (54.3) |
| No (did not attempt to define) 37 (45.7) |
| No (not correctly defined) 0 (0.0) |
| Unclear 0 (0.0) |

Table 5: Primary Estimand components by time period (n=81)

|  | January 2011 to August 2017^a^ | September 2017  to November 2019^b^ | December 2019 to December 2020^c^ |
| --- | --- | --- | --- |
| Population | n (%) | n (%) | n (%) |
| Was the Population correctly defined? | | | |
| Yes | 2/21 (9.5) | 15/37 (40.5) | 12/23 (52.2) |
| No (did not attempt to define) | 5/21 (23.8) | 8/37 (21.6) | 4/23 (17.4) |
| No (not correctly defined) | 14/21 (66.7) | 14/37 (37.8) | 7/23 (30.4) |
| Unclear | 0/21 (0.0) | 0/37 (0.0) | 0/23 (0.0) |
| If no (not correctly defined), why? |  |  |  |
| Estimand population defined as analysis population | 14/14 (100.0) | 14/14 (100.0) | 7/7 (100.0) |
| Treatment |  |  |  |
| Was the Treatment Condition(s) correctly defined? | | | |
| Yes | 14/21 (66.7) | 13/37 (35.1) | 9/23 (39.1) |
| No (did not attempt to define) | 7/21 (33.3) | 24/37 (64.9) | 14/23 (60.9) |
| No (not correctly defined) | 0/21 (0.0) | 0/37 (0.0) | 0/23 (0.0) |
| Unclear | 0/21 (0.0) | 0/37 (0.0) | 0/23 (0.0) |
| Endpoint |  |  |  |
| Was the Endpoint correctly defined? | | | |
| Yes | 11/21 (52.4) | 31/37 (83.8) | 17/23 (73.9) |
| No (did not attempt to define) | 10/21 (47.6) | 5/37 (13.5) | 6/23 (26.1) |
| No (not correctly defined) | 0/21 (0.0) | 1/37 (2.7) | 0/23 (0.0) |
| Unclear | 0/21 (0.0) | 0/37 (0.0) | 0/23 (0.0) |
| If no (not correctly defined), why? |  |  |  |
| Time point for assessment not given | 0/0 (0.0) | 1/1 (100.0) | 0/0 (0.0) |
| Summary measure |  |  |  |
| Was the Summary Measure correctly defined? | | | |
| Yes | 11/21 (52.4) | 21/37 (56.8) | 12/23 (52.2) |
| No (did not attempt to define) | 9/21 (42.9) | 14/37 (37.8) | 10/23 (43.5) |
| No (not correctly defined) | 1/21 (4.8) | 2/37 (5.4) | 1/23 (4.3) |
| Unclear | 0/21 (0.0) | 0/37 (0.0) | 0/23 (0.0) |
| If no (not correctly defined), why? |  |  |  |
| Authors did not state how the treatments will be compared, e.g. mean difference | 1/1 (100.00) | 2/2 (100.00) | 1/1 (100.00) |

^a^prior to release of guidance; ^b^consultation period; ^c^ guideline adopted

Table 6: Incorrect ICEs (n=81)

| Were any incorrect ICEs defined? n (%) |
| --- |
| Yes 8 (9.9) |
| No 73 (90.1) |
| Incorrect ICEs |
| Participants lost to follow-up / missing outcome data 6/8 (75.0)  Unspecified protocol violations 2/8 (25.0) |

Table 7: ICE1 - Treatment non-adherence / discontinuation where no reason specified (n=81)

| Was this listed as one of the ICEs? n (%) |
| --- |
| Yes 68 (84.0) |
| No 13 (16.0) |
| Was its handling correctly defined? |
| Yes 62/68 (91.2) |
| No (did not attempt to define) 3/68 (4.4) |
| No (not correctly defined) 3/68 (4.4) |
| Unclear 0/68 (0.0) |
| *If no (not correctly defined), why?* |
| Defined in terms of analysis, i.e., Intention-to-treat population (ITT) 3/3 (100.0) |
| *What strategy was used?* |
| Treatment policy 35/62 (56.5) |
| Hypothetical 15/62 (24.2) |
| Composite 10/62 (16.1) |
| Principal stratum 0/62 (0.0) |
| While-on-treatment 2/62 (3.2) |
| *Was the mechanism leading to hypothetical scenario given?* |
| Yes 4/15 (26.7) |
| No 11/15 (73.3) |
| Unclear 0/15 (0.0) |

Table 8: ICE2 - Treatment non-adherence / discontinuation due to adverse event (n=81)

| Was this listed as one of the ICEs? n (%) |
| --- |
| Yes 7 (8.6) |
| No 74 (91.4) |
| Was its handling correctly defined? |
| Yes 6/7 (85.7) |
| No (did not attempt to define) 0 (0.0) |
| No (not correctly defined) 0 (0.0) |
| Unclear 1/7 (14.3) |
| *What strategy was used?* |
| Treatment policy 1/6 (16.7) |
| Hypothetical 1/6 (16.7) |
| Composite 4/6 (66.7) |
| Principal stratum 0/6 (0.0) |
| While-on-treatment 0/6 (0.0) |
| *Was the mechanism leading to hypothetical scenario given?* |
| Yes 0 (0.0) |
| No 1/1 (100.0) |
| Unclear 0 (0.0) |

Table 9: ICE3 - Treatment non-adherence / discontinuation not due to an adverse event (n=81)

| Was this listed as one of the ICEs? n (%) |
| --- |
| Yes 5 (6.2) |
| No 76 (93.8) |
| Reason given for treatment non-adherence / discontinuation |
| Lack of efficacy 4/5 (80.0) |
| Wide disruptive event (COVID-19) 1/5 (20.0) |
| Was its handling correctly defined? |
| Yes 5/5 (100.0) |
| No (did not attempt to define) 0/5 (100.0) |
| No (not correctly defined) 0/5 (100.0) |
| Unclear 0/5 (100.0) |
| *What strategy was used?* |
| Treatment policy 0/5 (0.0) |
| Hypothetical 1/5 (20.0) |
| Composite 4/5 (80.0) |
| Principal stratum 0/5 (0.0) |
| While-on-treatment 0/5 (0.0) |
| *Was the mechanism leading to hypothetical scenario given?* |
| Yes 1/1 (100.0) |
| No 0/1 (0.0) |
| Unclear 0/1 (0.0) |

Table 10: ICE4 - Use of rescue therapy (n=81)

| Was this listed as one of the ICEs? n (%) |
| --- |
| Yes 29 (35.8) |
| No 52 (64.2) |
| Was its handling correctly defined? |
| Yes 24/29 (82.8) |
| No (did not attempt to define) 1/29 (3.5) |
| No (not correctly defined) 3/29 (10.3) |
| Unclear 1/29 (3.5) |
| *If no (not correctly defined), why?* |
| Defined based on method of analysis. 3/3 (100.0) |
| *What strategy was used?* |
| Treatment policy 8/24 (33.3) |
| Hypothetical 5/24 (20.8) |
| Composite 11/24 (45.8) |
| Principal stratum 0/24 (0.0) |
| While-on-treatment 0/24 (0.0) |
| *Was the mechanism leading to hypothetical scenario given?* |
| Yes 0/5 (0.0) |
| No 5/5 (100.0) |
| Unclear 0/5 (0.0) |

Table 11: ICE5 - Treatment switching (n=81)

| Was this listed as one of the ICEs? n (%) |
| --- |
| Yes 3 (3.7) |
| No 78 (96.3) |
| Was its handling correctly defined? |
| Yes 3/3 (100.0) |
| No (did not attempt to define) 0/3 (0.0) |
| No (not correctly defined) 0/3 (0.0) |
| Unclear 0/3 (0.0) |
| *What strategy was used?* |
| Treatment policy 1/3 (33.3) |
| Hypothetical 2/3 (66.7) |
| Composite 0/3 (0.0) |
| Principal stratum 0/3 (0.0) |
| While-on-treatment 0/3 (0.0) |
| *Was the mechanism leading to hypothetical scenario given?* |
| Yes 0/2 (0.0) |
| No 2/2 (100.0) |
| Unclear 0/2 (0.0) |

Table 12: ICE6 - Death (n=81)

| Was this listed as one of the ICEs? n (%) |
| --- |
| Yes 2 (2.5) |
| No 79 (97.5) |
| Was its handling correctly defined? |
| Yes 2/2 (100.0) |
| No (did not attempt to define) 0/2 (0.0) |
| No (not correctly defined) 0/2 (0.0) |
| Unclear 0/2 (0.0) |
| *What strategy was used?* |
| Treatment policy 0/2 (0.0) |
| Hypothetical 0/2 (0.0) |
| Composite 1/2 (50.0) |
| Principal stratum 0/2 (0.0) |
| While-alive 1/2 (50.0) |

Table 13: ICE7 - Other terminal event (n=81)

| Was this listed as one of the ICEs? n (%) |
| --- |
| Yes 1 (1.2) |
| No 80 (98.8) |
| Was its handling correctly defined? |
| Yes 1/1 (100.0) |
| No (did not attempt to define) 0/1 (0.0) |
| No (not correctly defined) 0/1 (0.0) |
| Unclear 0/1 (0.0) |
| *What strategy was used?* |
| Treatment policy 0/1 (0.0) |
| Hypothetical 0/1 (0.0) |
| Composite 0/1 (0.0) |
| Principal stratum 0/1 (0.0) |
| While-on-treatment 1/1 (100.0) |

Table 14: ICE8 - Other ICE (n=81)

| Were other ICEs listed? n (%) |
| --- |
| Yes 18 (22.2) |
| No 63 (77.8) |
| Description of ICE* |
| Did not receive study drug/study medication 14/20 (70.0) |
| Dose reduction or suspension of treatment 1/20 (5.0) |
| Ineligibility for follow-on study treatment 1/20 (5.0) |
| Participants who start a protocol prohibited medication/therapy 2/20 (10.0) |
| Change in background medication 1/20 (5.0) |
| Need for surgery 1/20 (5.0) |
| Was its handling correctly defined? |
| Yes 6/20 (30.0) |
| No (did not attempt to define) 7/20 (35.0) |
| No (not correctly defined) 7/20 (35.0) |
| Unclear 0/20 (0.0) |
| *If no (not correctly defined), why?* |
| Defined based on method of analysis. 7/7 (100.0) |
| *What strategy was used?* |
| Treatment policy 3/6 (50.0) |
| Hypothetical 0/6 (0.0) |
| Composite 2/6 (33.3) |
| Principal stratum 1/6 (16.7) |
| While-on-treatment 0 (0.0) |

*2 studies had 2 Other ICEs (n=20)
